# High Expression of LAMP3 Is a Novel Biomarker of Poor Prognosis in Patients with Esophageal Squamous Cell Carcinoma

**DOI:** 10.3390/ijms160817655

**Published:** 2015-07-31

**Authors:** Xiaoyu Liao, Yuanbin Chen, Deqing Liu, Fangfang Li, Xizhao Li, Weihua Jia

**Affiliations:** State Key Laboratory of Oncology in South China, Collaborative Innovation Center for Cancer Medicine, Sun Yat-Sen University Cancer Center, Guangzhou, China; E-Mails: liaoxiaoyu2010@163.com (X.L.); chen-yuanbin@hotmail.com (Y.C.); liudeq@sysucc.org.cn (D.L.); liff_2012@sina.cn (F.L.); lxz811@126.com (X.L.)

**Keywords:** LMAP3, esophageal squamous cell carcinoma, mRNA expression, DNA copy number

## Abstract

Lysosomal-associated membrane protein 3 (LAMP3), identified as a molecular marker of mature dendritic cells, is one of the LAMP family members. Its expression was induced by hypoxia, and was associated with hypoxia mediated metastasis in breast and cervical cancers. However, epithelial expression of LAMP3 and its prognostic value in esophageal squamous cell carcinoma (ESCC) is still unknown. In the current study, mRNA expression of LAMP3 in 157 ESCC tissues and 50 adjacent normal tissues was detected by quantitative real-time PCR (qRT-PCR). LAMP3 protein expression in 46 paired cancerous and normal tissues was detected by immunohistochemistry (IHC). Then, DNA copy number was examined to observe its potential correlation with mRNA expression. The results showed that both mRNA and protein expression level of LAMP3 was significantly higher in cancerous tissues compared with normal controls (*p* < 0.001). LAMP3 DNA copy number was amplified in 70% of ESCC tissues and positive correlated with mRNA expression (*p* = 0.037). Furthermore, patients with higher LAMP3 expression had worse overall survival (HR = 1.90, 95% CI = 1.17–3.09, *p* = 0.010) and disease-free survival (HR = 1.80, 95% CI = 1.18–2.74, *p* = 0.006). In conclusion, our results suggest that epithelial LAMP3 expression is an independent prognostic biomarker for ESCC.

## 1. Introduction

Esophageal squamous cell carcinoma (ESCC) is one of the most common cancer worldwide, especially in China. It is the fourth most common cause of cancer-related deaths [1,2]. ESCC is progressing rapidly and remains poor prognosis with a five-year overall survival rate ranging from 15% to 25% [3]. Wide spread lymph node metastasis and relatively frequent distant metastasis are the major factors determining its prognosis. Surgical resection is still a major therapeutic strategy for ESCC patients. To improve clinical outcome of patients with ESCC, novel molecular biomarkers used for early diagnosis, molecular target treatment, and prognosis prediction for ESCC patients have been widely investigated. It has been reported that aberrant expression of several important genes was associated with the poor prognosis of ESCC, such as p53, Bcl-2 and p300 [4,5].

Lysosomal-associated membrane protein 3 (LAMP3) was first isolated as a new gene, which specifically expressed in lung tissues and described as *TSC403* [6]. It codes a 416 amino acid protein. Followed study found that it is a molecular marker of mature dendritic cells, thereby named as *DC-LAMP* [7]. LAMP3 is the third member of the LAMP family proteins, a series of highly glycosylated type 1 integral membrane proteins. LAMP1 and LAMP2 are primarily located on the lysosomal membrane and are rarely expressed on the surface of normal cells [7,8]. However, previous study has observed that LAMPs protein could relocalize to the plasma membrane in some cancer cells [9]. Additional study has found that colon cancer cell lines exhibited an enhanced expression of LAMP1 and LAMP2 on the cell membrane and showed a stronger metastatic capacity [10]. These observations implicate that LAMPs may be important for the metastasis of malignant tumors.

Although the definite function of LAMP3 has not been identified, recent studies have identified that over expression of LAMP3 promoted metastasis of breast cancer cells [11,12]. In gastrointestinal cancer tissues, LAMP3 expression was not only elevated significantly compared with normal tissues, its high expression was also associated with poor overall survival in patients with gastrointestinal cancer, cervical cancer and breast cancers [11,13,14]. Furthermore, it has reported that upregulation of LAMP3 was associated with resistance to radiotherapy and chemotherapy [15,16]. It suggested that LAMP3 may be an important molecular marker for prognosis in cancers.

*LAMP3* is located on chromosome 3q27.1. Chromosome 3q is frequently amplified in several types of squamous cell carcinomas [17,18,19,20]. It has reported that chromosome copy number amplification at 3q was common in ESCC tissues and was associated with lymph node metastasis [20,21]. Dysregulation of genes in this region might play an important role in the progression and prognosis of cancers. For example, gene *EIF5A2* on 3q26 showed increased expression in over 40% of ESCC tissues, and its higher expression was significantly associated with lymph node metastasis [22]. Other 3q locus genes, including *PIK3CA*, *FGF12* and *TNF2*, have been identified as important genes in malignant tumors [19,23].

In this study, we detected epithelial LAMP3 expression by qRT-PCR and immunohistochemistry (IHC) in ESCC tissues and adjacent normal tissues. We analyzed the correlation between the expression level of LAMP3 and the clinicopathological characteristics in ESCC patients, as well as evaluated LAMP3 as a possible biomarker for ESCC prognosis.

## 2. Results

### 2.1. The Expression Level of LAMP3 in ESCC

**Figure 1:**
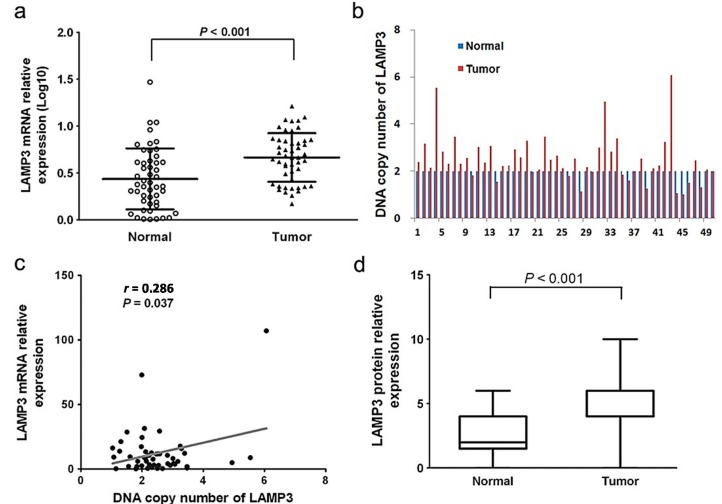
The expression level and DNA copy number of LAMP3 in ESCC tissues and adjacent normal controls. (**a**) LAMP3 mRNA expression level in 50 paired cancerous and normal tissues was detected by qRT-PCR. Triangles, the ESCC tumor tissue samples; circles, the adjacent normal tissue samples; (**b**) DNA copy number of LAMP3 in 50 paired cancerous and normal tissues was detected by qRT-PCR; (**c**) The correlation between LAMP3 DNA copy number and its transcriptional expression level in tumor tissues (*n* = 50); (**d**) LAMP3 protein expression level in 46 paired cancerous and normal tissues was detected by IHC.

**Figure 2:**
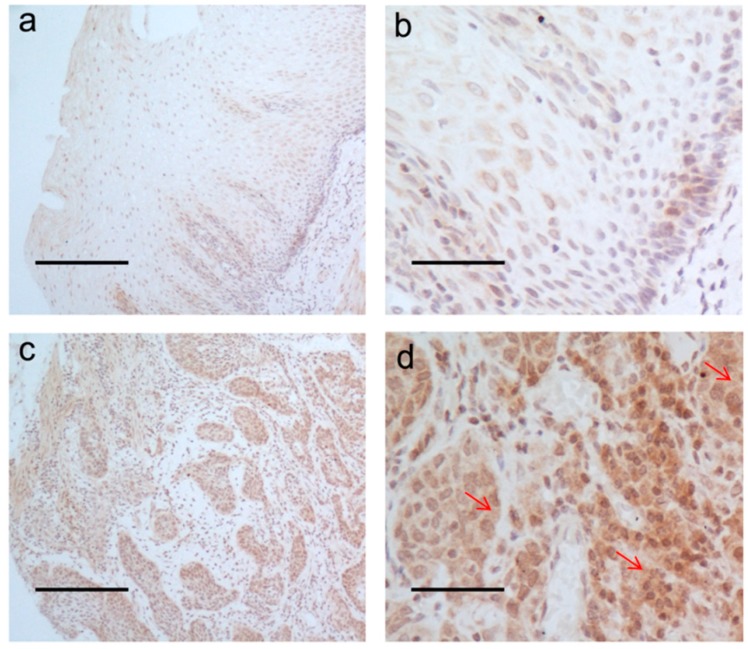
LAMP3 protein expression by IHC assay. (**a**,**b**) Normal esophageal mucosa showed low expression of LAMP3 protein; (**c**,**d**) High expression level of LAMP3 was observed in ESCC tissues. (**a**,**c**) 10×, scale bars: 200 μm; (**b**,**d**) 40×, scale bars: 50 μm. The red arrowheads point out the plasma membrane and cytoplasm area that are stained positive.

A total of 47 females and 110 males, aged from 34 to 80 years (mean age: 57 years) were enrolled in this study. The clinicopathological characteristics of the 157 patients are listed in Table 1. LAMP3 mRNA expression was detected by qRT-PCR in 157 ESCC tissues and 50 adjacent normal tissues. LAMP3 expression level in ESCC tissues was significantly higher than the paired normal controls (*p* < 0.001, Figure 1a). In normal epithelial cells and tumor cells, the positive immunostaining was mainly located at the plasma membrane and cytoplasm (Figure 2). The median score of ESCC tumors and their adjacent controls was 4 and 2 respectively. The cut-off value was the median score (4 scores) of ESCC tumors. High protein expression (score > 4) of LAMP3 was observed in 34.8% (16/46) of tumors and 10.9% (5/46) of paired controls. LAMP3 protein expression was also confirmed to be significantly higher in tumors when compared with paired normal tissues (*p* < 0.001, Figure 1d).

### 2.2. Overexpression of LAMP3 Was Correlated with DNA Copy Number Amplification

As LAMP3 is located on chromosome 3q27, a frequently amplified region in ESCC, we detected the DNA copy number of LAMP3 in 50 paired cancerous and adjacent normal tissues by qRT-PCR. As shown in Figure 1b, the LAMP3 DNA copy number was amplified in 70.0% (35/50) of ESCC tissues. We found that the correlation between LAMP3 mRNA expression and its DNA copy number was significant (*r* = 0.286, *p* = 0.037, Figure 1c). This result suggested that the DNA amplification of LAMP3 might result in its transcript over expression in a certain degree.

### 2.3. Association between LAMP3 Expression and Survival

In the 157 primary ESCC tissues, 51.0% (80/157) of cases were included in the LAMP3 high expression group, and the remaining cases were included in the LAMP3 low expression group. The expression level of LAMP3 in ESCC was not significantly correlated with gender, tumor size, tumor location, histologic grade and pathologic stage, but was for age (Table 1). Furthermore, the LAMP3 expression in ESCC was significantly correlated with alcohol drinking (*p* = 0.018). Among the 157 patients, the median observation period was 49.6 months (range: 3.6–106.7 months), and 78 patients were alive and 79 were deceased at the endpoint of follow-up. All of the 79 deaths during follow-up were from ESCC itself, rather than non-cancer reasons, such as angiocardiopathy or senile disease. The 5-year overall survival (OS) rate and 5-year disease-free survival (DFS) rate for the patients were 49.7% and 40.1%, with median survival time of 50.5 and 37.9 months, respectively.

**Table 1:** LAMP3 expression and clinicopathological characteristics in ESCC.

Variable	Cases	LAMP3 Expression		*p* Value ^a^
		Low (%)	High (%)	
**Gender**	-	-	-	0.304
Male	110	51 (46.4)	59 (53.6)	-
Female	47	26 (55.3)	21 (44.7)	-
**Age (years)**	-	-	-	0.003
<57	73	45 (61.6)	28 (38.4)	-
≥57	84	32 (38.1)	52 (61.9)	-
**Tumor Size (cm)**	-	-	-	0.063
<3.9	76	32 (42.1)	44 (57.9)	-
≥3.9	77	44 (57.1)	33 (42.9)	-
**Tumor Location**	-	-	-	0.063
Upper	12	2 (16.7)	10 (83.3)	-
Middle	102	52 (51.0)	50 (49.0)	-
Lower	43	23 (53.5)	20 (46.5)	-
**Histologic Grade**	-	-	-	0.344
G1	34	16 (47.1)	18 (52.9)	-
G2	79	43 (54.4)	36 (45.6)	-
G3	44	18 (40.9)	26 (59.1)	-
**T Status**	-	-	-	0.749
T1–T2	31	16 (51.6)	15 (48.4)	-
T3–T4	126	61 (48.4)	65 (51.6)	-
**Lymph Node Metastasis**	-	-	-	0.134
N0	68	38 (55.9)	30 (44.1)	-
N1	89	39 (43.8)	50 (56.2)	-
**TNM Stage**	-	-	-	0.095
I–IIa	59	34 (57.6)	25 (42.4)	-
IIb–IV	98	43 (43.9)	55 (56.1)	-
**Cigarette Smoking**	-	-	-	0.155
Never	53	30 (56.6)	23 (43.4)	-
Smoking	101	45 (44.6)	56 (55.4)	-
**Alcohol**	-	-	-	0.018
Never	92	52 (56.5)	40 (43.5)	-
Drinking	62	23 (37.1)	39 (62.9)	-

^a^ Pearson chi-squared test.

**Figure 3:**
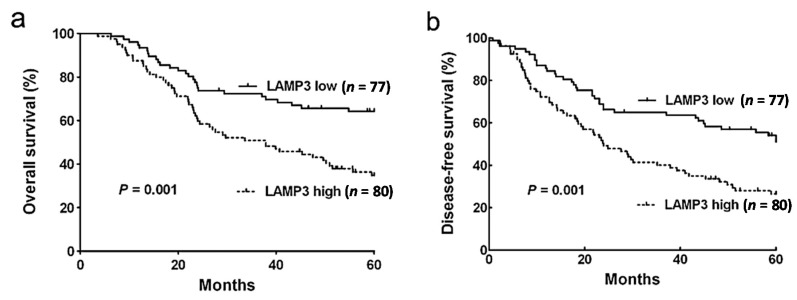
Kaplan-Meier survival curves for ESCC patients with high and low expression levels of LAMP3. (**a**) 5-year OS curves for 157 ESCC patients; (**b**) 5-year DFS curves for 157 ESCC patients. ESCC patients with high expression level of LAMP3 had poor OS and DFS (*p* = 0.001, log-rank test).

Patients with a higher expression of LAMP3 showed worse OS (*p* = 0.001, Figure 3a), which is consistent with the DFS analysis (*p* = 0.001, Figure 3b). Univariate analysis with Cox’s proportional hazard model showed that several clinicopathological characteristics were significantly associated with survival, including age, histologic grade and lymph node metastasis (Table 2 and Table 3). After being adjusted for these characteristics, the results of multivariate analysis suggested that LAMP3 expression was a significant independent prediction factor for OS (HR = 1.90, 95% CI = 1.17–3.09, *p* = 0.010) and DFS (HR = 1.80, 95% CI = 1.18–2.74, *p* = 0.006) in ESCC patients (Table 2 and Table 3).

**Table 2:** Univariate and multivariate survival analysis for overall survival according to the Cox regression model.

Risk Factors	Overall Survival			
	Univariate		Multivariate	
	HR (95% CI)	*p* Value	HR (95% CI)	*p* Value
LAMP3 Expression (Low/High)	2.22 (1.39, 3.55)	0.001 *	1.90 (1.17, 3.09)	0.010 *
Gender (Male/Female)	0.97 (0.60, 1.57)	0.892	-	-
Age (<57/57)	2.17 (1.35, 3.47)	0.001 *	1.66 (1.01, 2.72)	0.042 *
Tumor Size (<3.9/≥3.9 cm)	0.58 (0.37, 0.92)	0.022 *	0.64 (0.40, 1.02)	0.059
Tumor Location (Upper/Middle/Lower)	0.84 (0.56, 1.26)	0.399	-	-
Histologic Grade (G1/G2/G3)	1.69 (1.23, 2.33)	0.001 *	1.40 (0.99, 1.97)	0.055
T Status (T1–T2/T3–T4)	1.13 (0.64, 1.99)	0.671	-	-
Lymph Node Metastasis (N0/N1)	3.14 (1.90, 5.20)	0.001 *	2.66 (1.58, 4.48)	0.001 *

CI: confidence interval; HR: hazard ratio; * *p* < 0.05.

**Table 3:** Univariate and multivariate survival analysis for disease-free survival according to the Cox regression model.

Risk Factors	Disease-Free Survival			
	Univariate		Multivariate	
	HR (95% CI)	*p* Value	HR (95% CI)	*p* Value
LAMP3 Expression (Low/High)	2.00 (1.32, 3.03)	0.001 *	1.80 (1.18, 2.74)	0.006 *
Gender (Male/Female)	0.82 (0.53, 1.28)	0.387	-	-
Age (<57/57)	1.69 (1.12, 2.56)	0.013 *	1.48 (0.98, 2.26)	0.065
Tumor Size (<3.9/≥3.9 cm)	0.76 (0.51, 1.15)	0.199	-	-
Tumor Location (Upper/Middle/Lower)	0.81 (0.57, 1.15)	0.238	-	-
Histologic Grade (G1/G2/G3)	1.70 (1.27, 2.27)	0.001 *	1.42 (1.05, 1.92)	0.024 *
T Status (T1–T2/T3–T4)	1.26 (0.75, 2.13)	0.389	-	-
Lymph Node Metastasis (N0/N1)	2.58 (1.66, 4.01)	0.001 *	2.08 (1.31, 3.29)	0.002 *

CI: confidence interval; HR: hazard ratio; * *p* < 0.05.

## 3. Discussion

To our knowledge, this is the first study to analyze LAMP3 expression in human ESCC tissues and adjacent normal tissues by qRT-PCR and IHC methods. We observed that mRNA and protein expression of LAMP3 was significantly higher in ESCC tissues when compared with their adjacent normal controls, and LAMP3 expression was significantly positive correlated with its DNA copy number. Furthermore, in both univariate and multivariate analysis, the ESCC patients with higher expression level of LAMP3 had a worse overall survival and a worse disease-free survival. These results suggested that LAMP3 may be involved in the progression of ESCC.

LAMP3 belongs to the LAMPs family, which is characterized by high and complex levels of glycosylation [8,24]. Their expression have been identified in cancers, especially in relation to the metastasis of cancers [10,25,26]. LAMPs are the major carriers of sialyl-Lewisx antigens [27,28], which are important ligands for E-selectin. Through the interaction between sialyl-Lewisx and E-selectin, LAMPs expressed cancer cells can connect with E-selectins and adhere with vascular endothelial cells [29], which may be helpful in the metastasis of cancer cells. LAMP3 is localized at the lysosomal membrane under physiological conditions. It was identified as a molecular marker of mature dendritic cells (DCs) [7]. Liu and his colleagues reported that LAMP3 positive mature DCs were particularly dense in the margin of ESCC tissues [30]. However, LAMP3 expression has been observed in both DCs and epithelial cells in some cancers, and had a prognostic value in breast cancer [31,32]. In breast cancer cell lines, LAMP3 was induced by hypoxia *in vitro* and *in vivo*, and the correlation between mRNA expression level and lymph node metastasis implicated the hypothesis of a function of LAMP3 in the hypoxia-mediated metastasis in breast cancer [31]. In addition to this, the function of LAMP3 co-localization with hypoxic areas and regulating hypoxia-driven nodal metastasis was also observed in cervical cancer [12,14]. As the results listed in Table 1, high expression of LAMP3 was not significantly correlated with large tumor sizes (57.9% *vs.* 42.9%). Large ESCC tumors might induce hypoxia, and hypoxia could induce LAMP3 expression, so high expression of LAMP3 might be induced in large ESCC tumors. However, this result was not observed in our study. We think that the expression of LAMP3 in ESCC might also be regulated by other mechanism, such as DNA copy number variation (Figure 1c), microRNAs, and long non-coding RNAs regulation. In our study, high expression of LAMP3 was more frequent at the primary sites of patients with lymph node metastasis than in those without metastasis (56.2% *vs.* 44.1%), although not statistically significant, which might due to the small sample size (50 *vs.* 30). We need to collect more primary ESCC samples at different N statuses in the next step of research. Future *in vivo* and *in vitro* studies are needed to research the molecular mechanism of LAMP3 in tumorigenesis and metastasis of esophageal squamous cell carcinoma.

Furthermore, *LAMP3* is located at chromosome 3q, which is often amplified in cancer tissues. Several important oncogenes have been identified in this region, such as *PIK3CA* [33], *HTR* [34] and *EIF5A2* [35]. We have found that *LAMP3*, amplified in ESCC tissues, was associated with the prognosis of ESCC patients. We propose that *LAMP3* should be one of the candidate oncogenes at the amplified region in ESCC.

There are some limitations in our study. Firstly, as the sample size for detecting LAMP3 protein expression in tissues is small (*n* = 46), survival analysis cannot be performed using the protein expression level. Secondly, the number of patients is insufficient for subgroup analysis. For examples, there were only seven patients at M1 status and nine patients at T1 status. This limited number may lead to the result that expression level of LMAP3 was not associated with the survival and the clinicopathological characteristics in these subgroup patients.

In conclusion, we have demonstrated that LAMP3 expression level is an independent prognostic marker in esophageal squamous cell carcinoma. Future *in vivo* and *in vitro* studies are needed to research the molecular mechanism of LAMP3 in tumorigenesis and metastasis of esophageal squamous cell carcinoma.

## 4. Materials and Methods

### 4.1. ESCC Patients and Samples

A total of 157 ESCC patients underwent surgery at Sun Yat-Sen University Cancer Center (SYSUCC) in Guangzhou of Guangdong Province between April 2004 and December 2009, and were enrolled in this study. The criteria for included patients was as follows: (1) all cases were histologically diagnosed as ESCC; (2) patients did not receive radiotherapy or chemotherapy before complete surgical resection; (3) patients with non-curative resection or died of postoperative complications were excluded from this study; (4) patients’ clinical information, based on medical records, was collected, and follow-up data were collected via telephone interview. The clinical stage and histologic grade of the tumors were defined according to the sixth edition of TNM classification of the International Union Against Cancer (2002). The patients were comprised of 110 men and 47 women, with ages ranging from 34 to 80 years (mean age: 57 years). Esophageal tumor tissues and adjacent normal tissues were immediately frozen in liquid nitrogen after surgery and stored at −80 °C for subsequent extraction of DNA and RNA. Regarding cigarette smoking, ESCC patients who had smoked more than 100 cigarettes were defined as smokers. Alcohol drinking was defined as the consumption of all alcoholic beverages, including beer, wine, and liquor, which the patients regularly and consistently consumed during each time period [36]. This study was reviewed and approved by the Human Ethics Committee of SYSUCC. Informed consent was obtained from all enrolled patients.

### 4.2. Quantitative Real-Time PCR (qRT-PCR)

Total RNA was extracted using TRIzol reagent (Invitrogen, Carlsbad, CA, USA) following the manufacture’s protocol. Quality control of the RNA was completed by a bioanalyzer (Agilent 2100, Santa Clara, CA, USA). One microgram of total RNA from each sample was reversely transcribed by the PrimeScript^®^ RT reagent Kit with a gDNA Eraser (Takara, Dalian, China). Sequence data for LAMP3 was acquired from the UCSC Genome Browser Database (ID: 27074, NM_014398.3). LAMP3 mRNA relative expression was assayed by quantitative real-time PCR (qRT-PCR). The PCR system consisted of amplification primers LAMP3-F: 5′-CCTTCAAGTGCG TGAGTGAA-3′; and LAMP3-R: 5′-CCATAAGGCAGAGACCAACC-3′. The qRT-PCR reaction was set up in a reaction volume of 20 µL containing 10 µL Platinum^®^ SYBR Green qPCR Super Mix-UDG (Invitrogen), 0.5 µL forward primer (10 μm), 0.5 µL reverse primer (10 μm), 2 µL template, and 7 µL nuclease-free water. The PCR reaction was performed on a CFX96Touch (Bio-Rad, Hercules, CA, USA). All samples were performed in triplicate. The amplification was run with an initial denaturation for 5 min at 95 °C, followed by 40 cycles of denaturation at 95 °C for 15 s, annealing at 60 °C for 30 s, and extension at 72 °C for 15 s. The relative expression of LAMP3 was calculated with the following equation: relative expression = 2^−Δ*C*t^, Δ*C*_t_ = *C*_t_ (LAMP3) − *C*_t_ (GAPDH). GAPDH is the reference gene for normalization.

### 4.3. DNA Copy Number Analysis

Genomic DNA was extracted from the frozen tissues by a DNeasy Blood and Tissue Kit (Qiagen, Hilden, Germany) following the manufacture’s instructions. The DNA copy number of LAMP3 was examined by qRT-PCR as above. The primers included LAMP3-CNV-F: 5′-CCACACCCAACAACTCACAC-3′ and LAMP3-CNV-R: 5′-CTGGAAGGGTGGTCTGGTTA-3′. The DNA copy number of LAMP3 was calculated using the equation 2 × 2^−ΔΔ*C*t^, ΔΔ*C*_t_ = *C*_t_ (LAMP3) − *C*_t_ (reference) − *C*_t_(control) GRP15 and ZNF80 are reference genes for normalization.

### 4.4. Tissue Microarrays (TMAs) and Immunohistochemistry (IHC)

Formalin fixed, paraffin-embedded 46 paired primary ESCC tumor tissues and normal tissues, which were collected from Biobank of SYSUCC, were used for tissue microarrays (TMAs). TMA slides were deparaffinized in xylene, rehydrated with a serial concentration of ethanol, and incubated in 3% hydrogen peroxide for 10 min to quench endogenous peroxidase activity. For antigen retrieval, slides were heated in citrate buffer for 5 min. The tissue slides were incubated with rabbit anti-LAMP3 antibody (ab111090, Abcam, Cambridge, UK) overnight at 4 °C. Subsequently, the slides were incubated with HRP-conjugated mouse anti-rabbit immunoglobulin antibody. The nucleus was counterstained with hematoxylin. The IHC results were independently evaluated by two pathologists based on the following criteria: (1) staining intensity: zero (negative), 1 (weak), 2 (moderate) and 3 (strong); (2) percentage of positive cells: 1 (≤25%), 2 (25%–50%), 3 (50%–75%), 4 (>75%). The final IHC score was calculated by multiplying the staining intensity score by the score of positive cells (range 0–12). The samples were grouped into high and low groups by the median score (4 scores) of tumors as the cutoff value.

### 4.5. Statistical Analysis

The DNA copy number and mRNA expression level of LAMP3 in tumors were compared with adjacent normal tissues by paired *t*-test, while Wilcoxon signed-rank test was used for the comparison of LAMP3 protein levels between paired tumor and normal tissues. The correlation between expression level of LAMP3 and clinical characteristics was analyzed by chi-square test. Survival curves were estimated by Kaplan-Meier method. Hazard ratio (HR), 95% confidence interval (CI) and *p* values were calculated using multinomial logistic regression analysis. The cutoff value of LAMP3 mRNA expression high and low groups was determined by a Receiver operating characteristic (ROC) curve. All statistical tests were two-sided, and the level of statistical significance was defined as *p* < 0.05. Analyses were performed with STATA 10.0 (Stata Corp, College Station, TX, USA).

## 5. Conclusions

Our findings showed that both mRNA and protein expression level of LAMP3 was significantly higher in cancerous tissues compared with normal controls. LAMP3 DNA copy number was positive correlated with its mRNA expression. We have demonstrated that LAMP3 expression level is an independent prognostic marker in esophageal squamous cell carcinoma. Future in vivo and in vitro studies are needed to research the molecular mechanism of LAMP3 in tumorigenesis and metastasis of esophageal squamous cell carcinoma.
